# Dual Chamber Open Window Mapping and High‐Density Mapping for Atrioventricular Reentrant Tachycardia Associated With Atrioventricular Mahaim Fiber

**DOI:** 10.1002/joa3.70154

**Published:** 2025-07-24

**Authors:** Yuta Taomoto, Yuichi Ono, Ryota Ishida, Tatsuya Sakamoto, Kenichiro Otomo

**Affiliations:** ^1^ Department of Cardiology Ome Municipal General Medical Center Ome City Tokyo Japan

**Keywords:** 3D mapping, atrioventricular Mahaim fiber, atrioventricular reentrant tachycardia, catheter ablation, open window mapping

## Abstract

Dual‐chamber open‐window mapping (OWM) combined with high‐density mapping revealed early atrioventricular conduction on the lateral tricuspid annulus, consistent with a Mahaim fiber. Catheter ablation targeting the ventricular insertion site led to successful elimination of the accessory pathway and noninducibility of tachycardia.

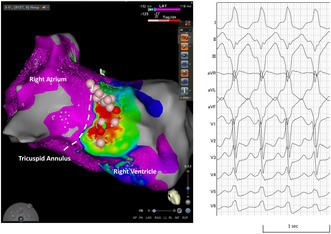

## 1 Spotlight

A 55‐year‐old man with a history of bronchial asthma presented with recurrent palpitations. During episodes of palpitations, an electrocardiogram (ECG) showed a wide QRS tachycardia with a heart rate of 150 bpm. At the time of his visit, tachycardia was still ongoing and was successfully terminated by slow intravenous administration of 5 mg of verapamil. After termination, no clear evidence of manifest ventricular pre‐excitation was observed on the ECG. A transthoracic echocardiogram revealed no significant abnormalities. Due to the recurrent symptomatic tachycardia, catheter ablation was performed.

**FIGURE 1:**
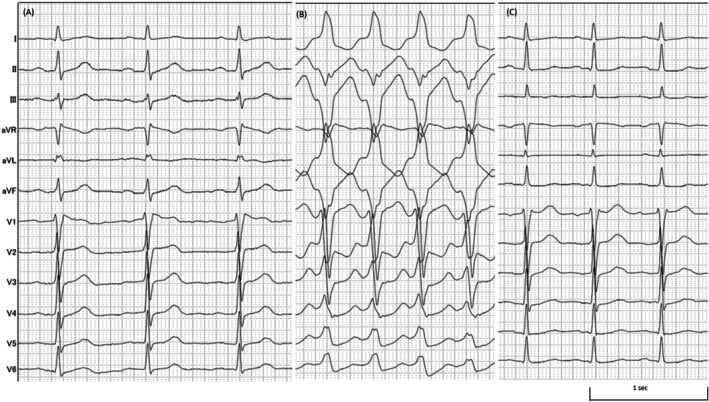
Twelve‐lead surface electrocardiogram. (A) Sinus rhythm with heart rate of 65 bpm. (B) Tachycardia with wide QRS complex, showing left bundle branch block morphology and cycle length of 439 ms. (C) Postoperative electrocardiogram.

**FIGURE 2:**
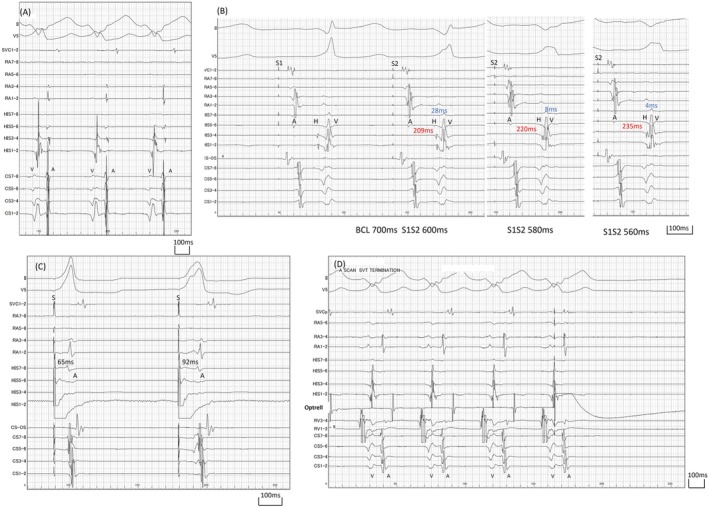
Intracardiac electrogram findings. (A) Intracardiac electrogram during tachycardia. The earliest site of atrial activation during ventriculoatrial conduction was located at the His bundle region. (B) Atrial pacing with a basic cycle length of 700 ms. Single premature atrial contractions were introduced at intervals of 600, 580, and 560 ms. The red text indicates the atrioventricular interval; the blue text indicates the His–ventricular interval. (C) During para‐Hisian pacing, no accessory pathway conduction was observed in the ventriculoatrial direction. (D) A single premature stimulus delivered from the right atrial lateral wall using the OPTRELL catheter during the atrial septal refractory period resulted in the termination of tachycardia without ventricular capture. A, atrial potential; BCL, basic cycle length; CS, coronary sinus; H, His bundle potential; RA, right atrium; S, stimulus; SVC, superior vena cava; V, ventricular potential.

Upon arrival at the cardiac catheterization laboratory, the patient was in sinus rhythm (Figure 1A). The clinically observed tachycardia recurred spontaneously during placement of the intracardiac electrode catheter (Figure 1B). During sinus rhythm, the atrial to His (AH) interval was 108 ms. The interval between the His potential and the ventricular electrogram recorded by the linear electrode catheter placed at the His bundle was 61 ms, while the interval between the His potential and the ventricular electrogram at the right ventricular apex was 51 ms, indicating that the right ventricular apex was activated earlier than the ventricular activation recorded at the His bundle (Figure S1). During tachycardia, the intracardiac electrogram showed a tachycardia cycle length of 439 ms, with the earliest ventriculoatrial activation noted at the His bundle region (Figure 2A).

Recently, dual‐chamber open‐window mapping (OWM) has been reported as an effective strategy for catheter ablation of accessory pathways [1]. Since the tachycardia recurred easily, dual‐chamber OWM was performed using the OPTRELL mapping catheter (Biosense Webster Inc., Diamond Bar, CA). Mapping was conducted using the CARTO 3 mapping system (Biosense Webster Inc., Diamond Bar, CA), with the window of interest set based on the ventricular potential, ranging from −215 to +222 ms. Annotations were automatically acquired using the −dp/dt parameter and were manually verified.

**FIGURE 3:**
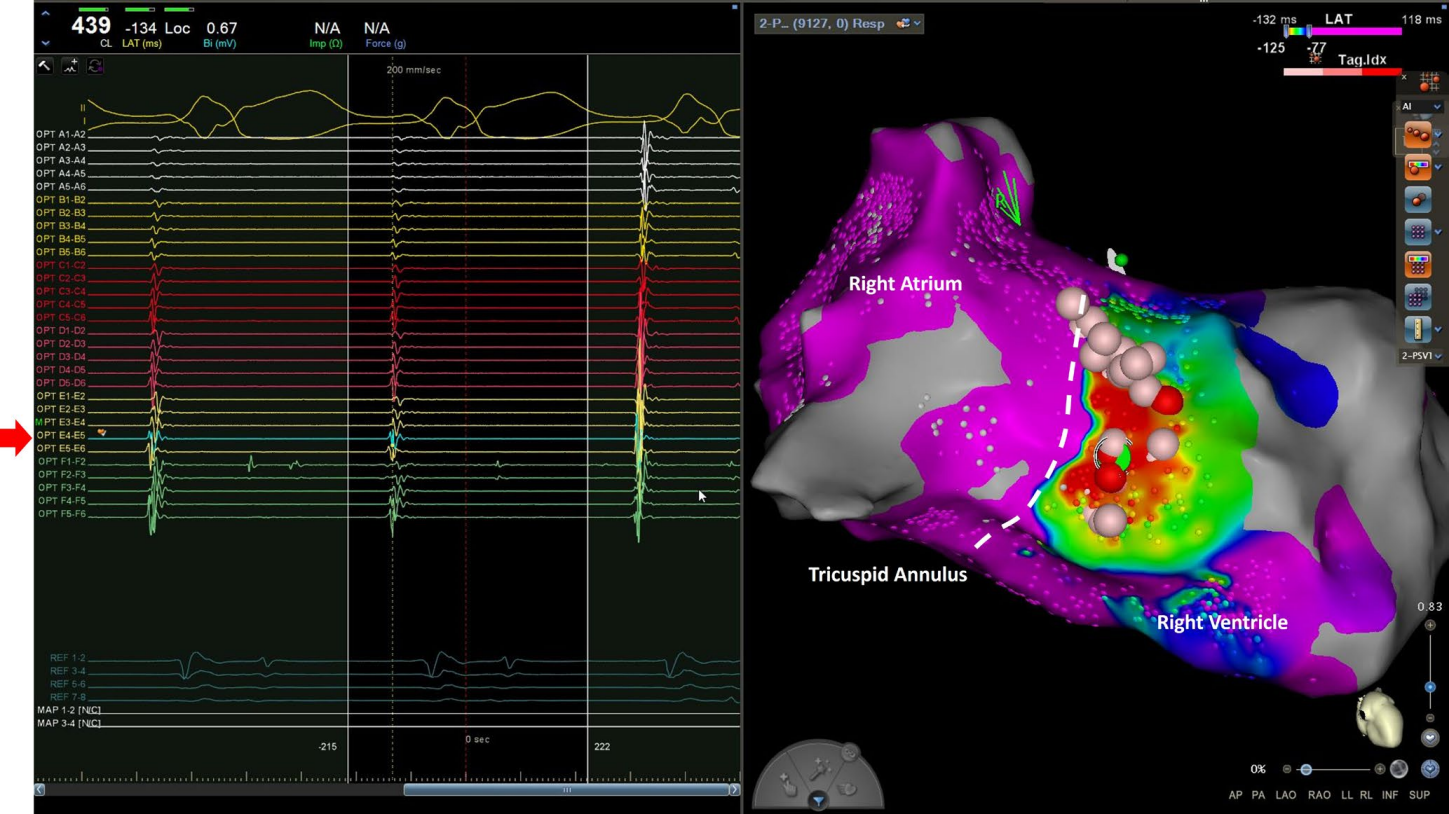
Dual‐chamber open‐window mapping during tachycardia using the CARTO system. The right image shows a view from the caudal side. The initial ablation site, where tachycardia was successfully terminated, is marked with a green tag. The corresponding electrogram at this site is shown in the left panel (red arrow). Red and pink tags indicate additional ablation sites. The tricuspid annulus was defined as the region where atrial and ventricular potentials were separated and where a white line was observed at a lower threshold of 20% in the early‐meets‐late algorithm (Figure S2). The dotted line indicates the anatomical location of the tricuspid annulus.

Three‐dimensional mapping revealed no evidence of continuous atrioventricular conduction through annulus such as Kent bundle. However, early excitation from the atrium to the ventricle was detected on the lateral ventricular wall adjacent to the tricuspid annulus, which was consistent with Mahaim fiber (Figure 3, Figure S2).

Subsequently, while terminating the tachycardia, electrophysiological testing was performed. During sinus rhythm, atrial extrastimuli were delivered, demonstrating shortening of the His–ventricular interval and decremental conduction of the atrioventricular interval (Figure 2B). Ventriculoatrial conduction occurred only through the His bundle, and no accessory pathway was identified using para‐Hisian pacing (Figure 2C). During tachycardia, the introduction of an extrastimulus from the lateral wall of the right atrium during atrial septal refractoriness resulted in termination of the tachycardia without ventricular capture (Figure 2D).

**FIGURE 4:**
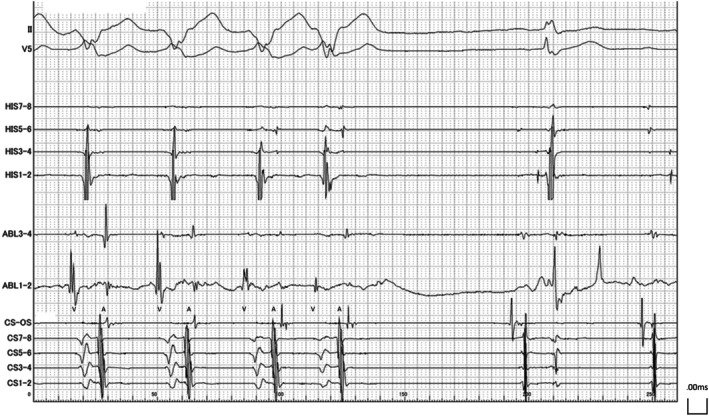
Intracardiac electrogram during ablation targeting the earliest ventricular activation site (marked in red in Figure 3). A, atrial potential; V, ventricular potential.

Based on these findings, the diagnosis was antidromic atrioventricular reentrant tachycardia (AVRT) mediated by atrioventricular Mahaim fiber [2]. No Mahaim potential was observed on the atrial side during mapping. Therefore, ablation was performed at the ventricular insertion site, guided by the earliest ventricular activation during tachycardia [3]. Thermocool Smarttouch SF Catheter (Biosense Webster Inc., Diamond Bar, CA) was used. The initial application with 35 W at this site resulted in tachycardia termination (Figure 4). However, the atrioventricular interval of the potential recorded by the ablation catheter changed just prior to termination, suggesting that the tachycardia may have been interrupted by a premature ventricular contraction rather than a successful elimination of the accessory pathway.

Additional applications were delivered to adjacent area. During pacing from the right atrial lateral wall, intermittent conduction through the accessory pathway was observed. Further ablation lesions were applied to surrounding regions. After a total of 30 ablation applications, atrioventricular conduction via the Mahaim fiber was completely eliminated. Following the administration of isoproterenol, tachycardia induction was attempted using continuous atrial pacing and extrastimulation; however, no further tachycardia could be induced, and the procedure was concluded. The postoperative electrocardiogram did not clearly show right bundle branch block (Figure 1C).

## 2 Discussion

In conventional activation mapping, precise annotation of atrial, accessory pathway, and ventricular potential is required to identify the target for ablation, making the process complex and time‐consuming. In contrast, OWM allows for the omission of manual annotation, enabling rapid estimation of the reentrant circuit and clear visualization of the ventricular insertion site.

In the present case, the OWM findings were helpful in supporting the diagnosis of AVRT related to atrioventricular Mahaim fiber, as the excitation bypassed the annulus and entered the ventricular myocardium, unlike in typical Kent bundle cases where conduction occurs through the annulus. However, these OWM findings alone were not sufficient to definitively diagnose AVRT mediated by atrioventricular Mahaim fiber, and comprehensive conventional electrophysiological evaluation remains essential.

Regarding Mahaim fiber, they exhibit an arborized structure, branching in detail and connecting to the ventricular myocardium [4]. The present case showed findings consistent with this, with simultaneous excitation over a broad area on the ventricular side.

Although detailed analysis using conventional mapping techniques has been reported to increase the likelihood of identifying Mahaim potential along the accessory pathway [5], that potential is absent in approximately one‐third of cases involving atrioventricular Mahaim fiber [4]. Consequently, while ablation is commonly guided by the identification of the Mahaim potential, targeting the ventricular insertion site represents an alternative strategy.

In the present case, electrogram assessment was performed using a multipolar electrode catheter, enabling high‐resolution evaluation with small electrodes. Nevertheless, Mahaim potential was not observed. Although suboptimal catheter‐tissue contact or incomplete mapping cannot be entirely excluded, the application of the OWM technique facilitated rapid and reliable estimation of the arborization extent based on the activation pattern. From a therapeutic standpoint, although multiple attempts were required before successful ventricular attachment ablation based on OWM, it is difficult to conclude that OWM offers a clear advantage over conventional mapping techniques. Nevertheless, this approach proved valuable in delineating the target region for ablation at the ventricular insertion site.

## 3 Conclusion

In conclusion, OWM was a useful technique for catheter ablation of AVRT involving atrioventricular Mahaim fiber. In the absence of detectable Mahaim potential on the atrial side, excitation did not conduct through the annulus as seen in typical Kent bundle cases, but instead entered the ventricular myocardium by skipping across the annulus (Video S1). This conduction pattern helped differentiate the Mahaim fiber from other pathways. Furthermore, the OWM results were helpful in determining the area for ventricular ablation, making it useful in that regard as well.

## Ethics Statement

The authors have nothing to report.

## Conflicts of Interest

The authors declare no conflicts of interest.

## Supporting information


Figure S1.

Figure S2.

Video S1.

## Data Availability

The authors have nothing to report.
