# Supplementary figures and images for: Dual Chamber Open Window Mapping and High‐Density Mapping for Atrioventricular Reentrant Tachycardia Associated With Atrioventricular Mahaim Fiber
Source: J Arrhythm. 2025 Jul 24;41(4):e70154. doi: 10.1002/joa3.70154 (PMC12289533; doi:10.1002/joa3.70154)

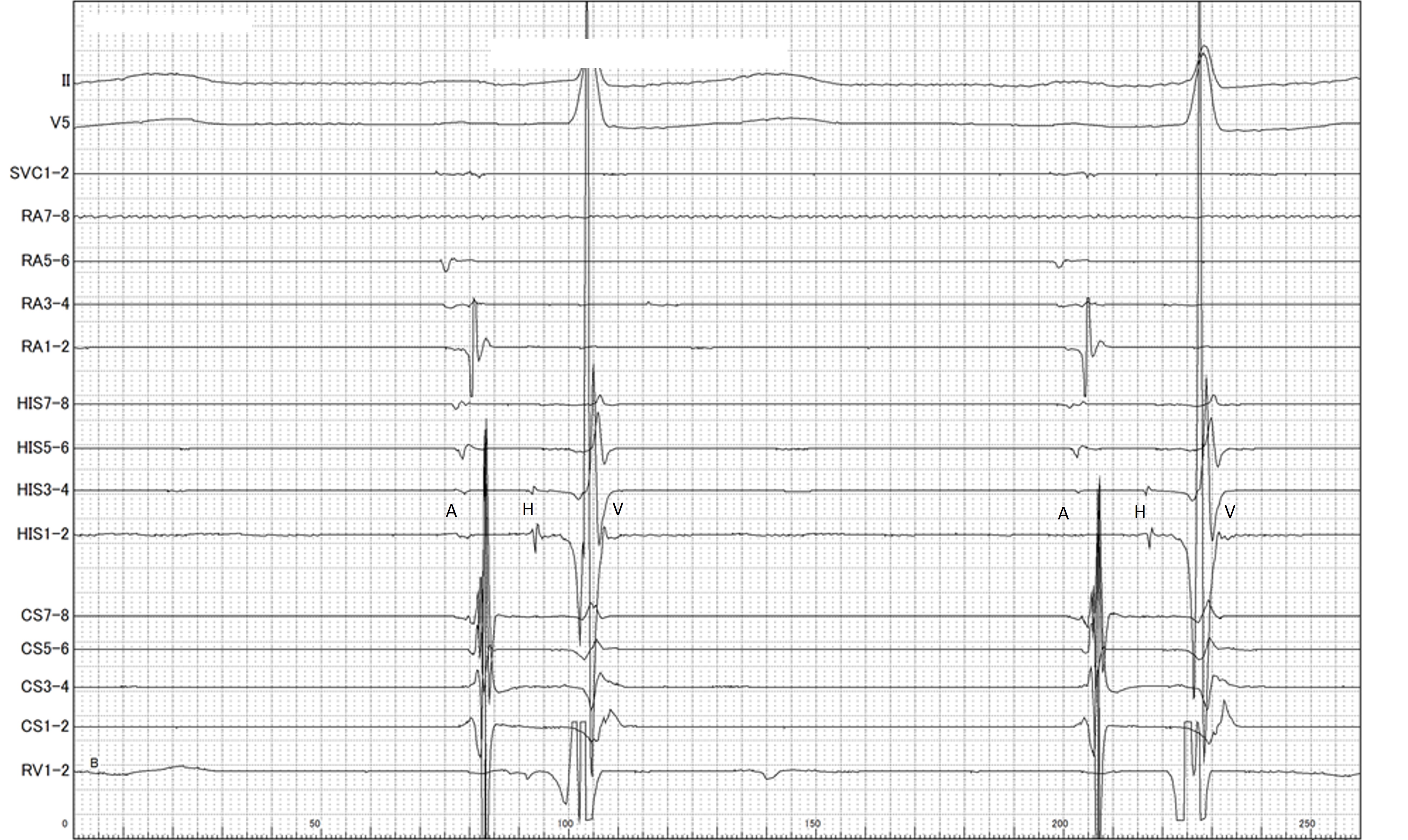

Supplement: Supplementary file 1 — Figure S1. [file JOA3-41-e70154-s001.jpg]

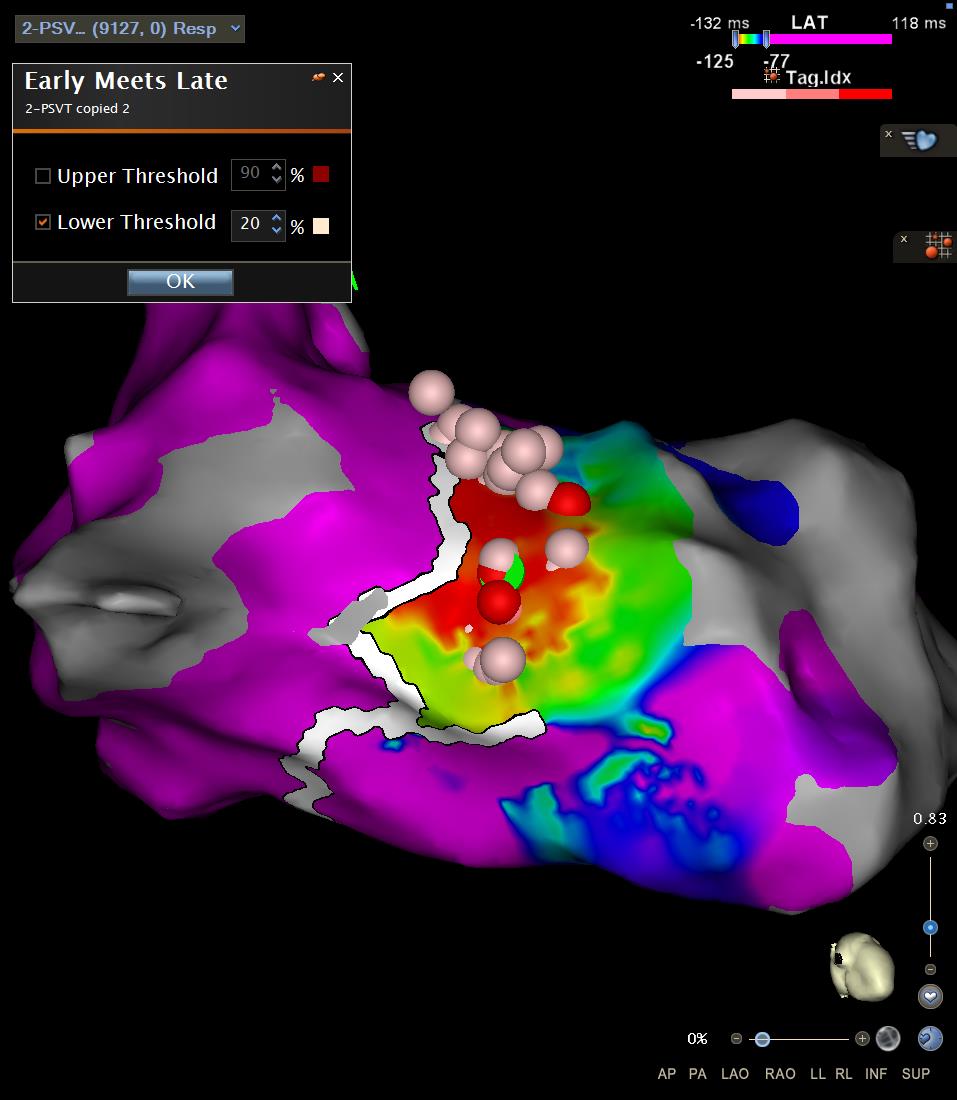

Supplement: Supplementary file 2 — Figure S2. [file JOA3-41-e70154-s003.jpg]
